# A Novel Approach to Railway Track Faults Detection Using Acoustic Analysis

**DOI:** 10.3390/s21186221

**Published:** 2021-09-16

**Authors:** Rahman Shafique, Hafeez-Ur-Rehman Siddiqui, Furqan Rustam, Saleem Ullah, Muhammad Abubakar Siddique, Ernesto Lee, Imran Ashraf, Sandra Dudley

**Affiliations:** 1 Faculty of Computer Science and Information Technology, Khawaja Fareed University of Engineering and Information Technology, Rahim Yar Khan 64200, Pakistan; rahmanshafique47@gmail.com (R.S.); furqan.rustam1@gmail.com (F.R.); saleem.ullah@kfueit.edu.pk (S.U.); 2 Department of Computer Science and Information Technology, Ghazi University, Dera Ghazi Khan 32201, Pakistan; Abubakar.ahmadani@gmail.com; 3 Department of Computer Science, Broward College, Broward Count, FL 33332, USA; 4 Department of Information and Communication Engineering, Yeungnam University, Gyeongsan 38541, Korea; imranashraf@ynu.ac.kr; 5 School of Engineering and Design, London South Bank University, London SE1 0AA, UK; dudleyms@lsbu.ac.uk

**Keywords:** railway track inspection, acoustic signals analysis, railway track cracks detection, machine learning, deep convolution neural networks, logistic regression

## Abstract

Regular inspection of railway track health is crucial for maintaining safe and reliable train operations. Factors, such as cracks, ballast issues, rail discontinuity, loose nuts and bolts, burnt wheels, superelevation, and misalignment developed on the rails due to non-maintenance, pre-emptive investigations and delayed detection, pose a grave danger and threats to the safe operation of rail transport. The traditional procedure of manually inspecting the rail track using a railway cart is both inefficient and prone to human error and biases. In a country like Pakistan where train accidents have taken many lives, it is not unusual to automate such approaches to avoid such accidents and save countless lives. This study aims at enhancing the traditional railway cart system to address these issues by introducing an automatic railway track fault detection system using acoustic analysis. In this regard, this study makes two important contributions: data collection on Pakistan railway tracks using acoustic signals and the application of various classification techniques to the collected data. Initially, three types of tracks are considered, including normal track, wheel burnt and superelevation, due to their common occurrence. Several well-known machine learning algorithms are applied such as support vector machines, logistic regression, random forest and decision tree classifier, in addition to deep learning models like multilayer perceptron and convolutional neural networks. Results suggest that acoustic data can help determine the track faults successfully. Results indicate that the best results are obtained by RF and DT with an accuracy of 97%.

## 1. Introduction

Railways are the lifeline of countries, especially developing countries like Pakistan, and serve the public’s transportation needs, as well as being the backbone for trade and supply chains. Over the years, the railway market has grown stronger, offering greater prospects for the public and the country’s economy. As reported in [1], an increase of 1.3% to 2.4% in the annual growth of the railway industry was observed between 2016 to 2018. However, for the uninterrupted operation of railway trains and the safety of travelers, high-performance railway operations need to be ensured. The public, including school children, busy commuters and tourists, travel on trains and their safety is compromised if railway tracks are not appropriate for regular operations. Similarly, cargo safety and reliability are pivotal for the supply chain and require fault-free and tolerant railway tracks. Since mechanical and physical wear and tear may occur over time, regular inspections are required to minimize the derailing of trains.

Pakistan is a country where a large number of people travel by train with an estimated 70 million doing so from 2018 to 2019 [2]. However, several major accidents have taken place over the past few years with high human and financial loss. Such accidents occur due to human error and railway tracks wearing out. According to the annual reports by Pakistan Railways [3], 127 accidents were registered between 2013 and 2020 due to trains derailing due to railway track faults. In 2013, a total of 22 trains, including 13 passenger trains and nine goods trains, were derailed. Another 16 passenger trains and 22 goods trains were derailed in 2014, the maximum for any state. In 2015, 37 passenger trains and 37 goods trains had accidents. According to a report on train derailing accidents for the years 2018–2019, on 16 September 2018, nine bogies were derailed near Attock and 22 passengers were badly injured [4]. On 27 September 2018, near Peshawar, a freight train was derailed, overturning 11 bogies. On 9 June 2019, 23 bogies of a Karachi-bound freight train were derailed near Sukkur.

Railway tracks need proper and timely maintenance; if they fail, it can have a major impact on train operations [5]. The identification of cracks is important for running the system efficiently. In order to reduce the negative impacts, the feasibility of a low-cost automated traditional cart System capable of monitoring the health of the railway track needs to be developed and assessed, in order to help with the efficient and accurate diagnosis and maintenance of tracks so as to avoid accidents. To minimize human error, automated fault detection of the railway track system is mandatory.

For the continuous operation of railway trains with a higher level of safety and reliability, railway track condition monitoring is of significant importance where railway tracks are continuously inspected to find and repair cracks. However, monitoring the hundreds of thousands of miles of railway track requires both a substantial amount of money and manpower. Even so, human inspection is prone to error and manual inspection is tedious and biased. For railway track inspection in Pakistan, currently, a railway cart is used where human experts manually inspect the track and find where the repairs are needed. Owing to the importance of track inspection, this study presents and contributes a smart automated cost-effective track conditions inspection method and makes the following contributions:

- An automatic railway track inspection system is presented which can discriminate three types of track condition including wheel burnt, superelevation, and normal track. The intended system triggers an alarm if it detects a defect. Capabilities can be added such as fault location and integration with IoT for remote fault detection understanding, enabling hot spot identification and reasoning;
- A dataset is collected spanning 1 year of collection, where an ECM-X7BMP microphone is used to collect the acoustic signal. The Mel-frequency cepstrum coefficients (MFCC) [6] features from the acoustic signals are later used with different classifiers for the automatic detection of track faults. The scope of this work is confined to detecting railway track faults using acoustic analysis;
- Two well-known machine learning classifiers, logistic regression (LR) and support vector machines (SVM), are used, as well as two deep learning approaches including multilayer perceptron (MLP) and convolutional neural network (CNN) for the automatic detection of railway track faults. The performance is evaluated using accuracy, precision, recall, and F1 score.

The rest of the paper is structured as follows. The background on the nature of different cracks in railway tracks and important research on identifying such faults are provided in Section 2. The data collection procedure, apparatus used for the data collection, and proposed research methodology are presented in Section 3. Section 4 contains the results and discussions while the conclusion is given in Section 5.

## 2. Background and Literature Review

**Figure 1:**
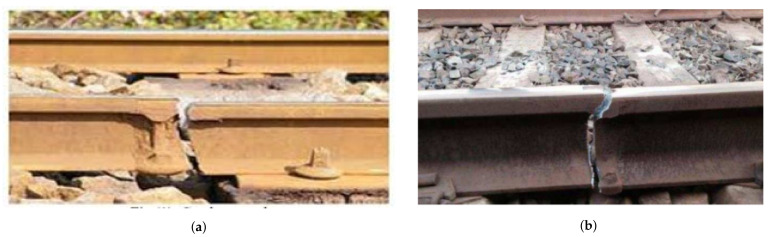
Samples of faults on railway tracks, (**a**) Destroyed patch [7]; (**b**) Partial crack [8]. Such faults can happen due to excessive loads, and the influence of cold and hot weather.

Because manually driven approaches are insufficient to monitor the health of tracks systematically, robustly, regularly, and uniformly due to human error, the automatic detection and monitoring of tracks’ faults/cracks is very important. However, an understanding of common problems related to railway tracks is crucial. Major railway track faults can be categorized into wheel burn, ballast issues, superelevation, and loose nuts and bolts. Figure 1a,b shows some examples of the cracks found on railway tracks in Pakistan. Such cracks appear due to several factors such as wear and tear due to the long use of the track without maintenance, overheating, displacement of supporting basement, and train overload, and so forth.

**Figure 2:**
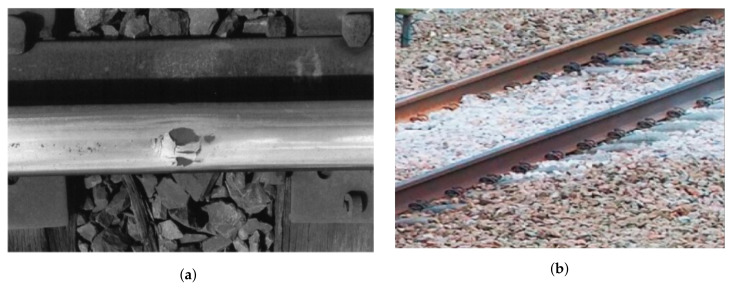
Wheel burn and ballast issue on railway track, (**a**) Wheel burnt issue on railway track [9]; (**b**) Week and expired ballast issue [10].

Wheel burn on a track appears either because of a jammed wheel as shown in Figure 2a or a locomotive jumping due to an imbalanced ballast. Similarly, an expired or weak ballast issue is shown in Figure 2b. A track ballast forms the trackbed upon which railroad sleepers (ties) are laid, packed below and around the ties bearing the load from the railroad sleepers to facilitate not only water drainage but also to dampen down vegetation that might interfere with the track’s structure.

Super Elevation arises when the outer rail of a track becomes higher than the desired elevation. The outer rail is normally set higher than the inner rail for a curved track. Most counties achieve the desired level of positive cant by raising the outside rail to a certain level, which is called superelevation.

**Figure 3:**
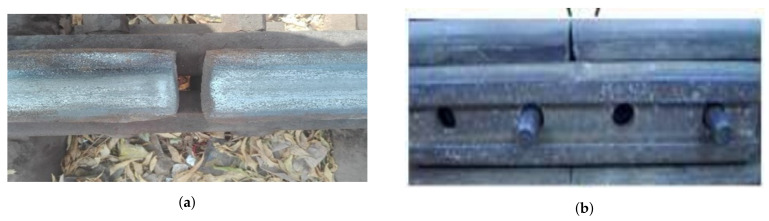
Surface and nuts and bolts problems of a railway track: (**a**) Damage of surface of rail head due to super elevation issue; (**b**) Absence of nuts and bolts [11].

Railway fish plates and fish bolts are a joint bar, a metal bar that is bolted to the ends of two rails to join them together in a track, and are the common rail connection parts. They are used to maintain the strength and stiffness of the joint for uniform elasticity. Most often, two railway fish plates are fixed on either side of the rail waist. Missing any one of the plates is usually due to missing nuts or bolts. This is also one of the main causes of train derailments. The superelevation problem is shown in Figure 3a while Figure 3b exhibits the nuts and bolts problem.

Acoustic analysis has the potential to distinguish and recognize sounds based on pitch, energy, sound entropy, and spectral analysis. The rapid growth and miniaturization of sensors and electronics equipment has made it ubiquitous and available on the market [12]. Researchers have shown its capability to classify defects in machines by their acoustic signature. Hence, this study leverages acoustic signals to detect and classify faults in Pakistan’s railway tracks.

An obvious reason to perform an inspection of railway tracks is to avoid train accidents and save human lives. For this purpose, periodic and regular inspection of railway tracks is of paramount importance. Track defects and non-compliance, if not spotted early, ultimately lead to stark consequences such as train derailments and loss of life. However, a human inspection of hundreds of thousands of miles of track is cumbersome, time-consuming, laborious, and subject to human error. Consequently, different automated approaches have been proposed to ease human effort and increase efficiency.

Track detection based on computer vision is becoming more popular among researchers. The use of drones instead of a moving wagon may provide even more cost-effective track monitoring. For example, the authors of [13] proposed a novel approach to computing gauge measurement using drone imagery and the health of the track was determined by applying computer vision techniques to the drone data. Da-Jiang Innovations (DJI) Phantom 3, professionally equipped with 4k camera and Sony sensors, is used for data acquisition. Images are taken at 29.76768000 and 78.01000000 Latitude and longitude respectively over a predefined path and images are automatically captured at continuous intervals. The images are converted into hue, saturation, value (HSV) color space to minimize the illumination effects caused by different weather conditions, followed by a Gaussian smoothing filter to reduce the noise. It is observed that the railway tracks have a purple/pinkish hue, so to obtain fine object (track) detection, all hues between the range of cyan and magenta are extracted by applying multiple threshold masks. Morphological operations are used to remove all connected pixels below a threshold value and subsequently, a Canny edge detector is applied for obtaining accurate results.

Railway track monitoring data are available but not all anomalies can be analyzed using image processing, for example, vegetation overgrowth and sun kinks are rare and difficult to find. These can be generated manually using tools like paint, but it can be a very cumbersome, labor-intensive process. Hence, if synthetic data can be generated for the anomalies mentioned above, it can ease the training process and reduce the problem of over-fitting. The study [14] performs a computer vision-oriented experiment using a camera that captures an image at 30 frames per second. It is mounted on a locomotive aiming for a consistent steady image for real-time railway track fault detection. The Inception V3 model is applied on the Image net dataset to fine-tune for a binary class classification. For vegetation overgrowth, the model generalizes well on actual vegetation images. A sun kink classifier can classify professionally simulated sun kink videos with a precision of 97.5%.

A visual-based track inspection system (VTIS) is attributed with a high speed, low cost, and attractive performance and is regarded as the most appealing track surface defect detection technique. Study [15] presents a VTIS system using a multiphase deep learning-based rail surface anomaly detection and classification technique called TrackNet. The study adopts CNNs, such as ResNet and DenseNet, as the baseline techniques for performance comparison with the proposed TrackNet. Results indicate the proposed system performs better than the baseline approaches. Another vision-based approach for track inspection and fault detection is presented in [16]. The input image is decomposed by a Gabor filter and texture features are extracted using segmentation-based fractal texture analysis (SFTA). The AdaBoost classifier is used to classify the track faults. Study [17] worked on the detection of cracks and missing fasteners in railway lines using the structure topic model (STM). The study proposed an effective vision-based automatic rail inspection system to detect the presence or absence of sleepers or fasteners, by inspecting real-time images acquired by a digital camera installed under a diagnostic train.

The authors designed a prototype in [18] that detects cracks using an Arduino mega powered by solar panels along with a LASER source. In addition, avalanche photodiodes (APD) and vibration sensors are used to detect cracks. A global positioning system (GPS) is also implemented to provide the exact location of the detected crack. The study [19] proposed an automated fault detection system consisting of different sensor modules mounted on a moving robot. Faults addressed in this study are discontinuity, obstacles on the track, absence of nuts and bolts, and misalignment. The sensors layer includes an infrared (IR) sensor, a limit switch [20] and ultrasonic sensors that are incorporated with an LPC 1768 ARM microcontroller. Upon the detection of any of the faults mentioned above, the localization along with fault type is sent to the control room by using the GSM module. Similarly, [21] designed a model robust railway crack detection scheme (RRCDS) to address the faults on tracks using IR sensors that detect cracks on the railway track. Existing manual systems are inefficient at monitoring the near-surface cracks precisely and are inappropriate for use in tunnels. Thus, to maintain safety standards, an economical and lower power PRCDS is presented in [22]. RRCDS is comprised of two IR sensors; an Arduino board coupled with Bluetooth is mounted at the front end of the inspection robot to monitor the track. This system automatically detects a faulty railway track without any human interference. It also tracks the GPS location of the track.

Early failure detection is critical for maintenance and to aid timely replacements to avoid accidents. [23] proposed a system for the early detection and diagnosis of faults in railway points using acoustic analysis. Dataset collection was performed by an NS-AM type railway point machine equipped with audio sensors for data collection. Faults, such as ice obstruction, ballast obstruction, and slackened nuts, were analyzed in this study. Two different experiments were carried out, one for fault detection on the whole dataset and the other for fault classification. The model evaluation shows an accuracy of 94.1%.

The authors performed an investigation regarding the detection of multiple types of fastener damage in [24]. A fastener is a place on the track where the track is fastened with the tie. An automated vision-based railway inspection system is proposed that uses SVM, AdaBoost, and likelihood algorithms for the detection of tracks and sleepers. Along the same lines, [25] proposed a railway track derailment inspection system for the automated visual inspection of railroad tracks, which detects faults from prerecorded videos. To detect the fault, spectral estimation and signal processing methods are utilized. The scope of the work [25] is confined to the localization of rail defects, ballast, tie and tie plate, and the localization of spikes, tie plate holes, and anchors.

Real-time rail track detection and adaptability is paramount to prevent human, goods, and assets losses. Yongzhi Min et al. [26] devised a real-time visual portable machine vision inspection system for track defects. It is equipped with an acoustic emission sensor and a passive infrared (PIR) sensor for the detection of cracks. An acoustic emission sensor is used to detect cracks on a track and a PIR sensor is used to check for the presence of human or animal bodies on the track. Written in LabVIEW environment, the system accesses high-quality images from a light source environment by adding the hoods and LED auxiliary light source in the image acquisition equipment in the first stage. In the second stage, the H value of the color image is used directly to extract the original image, which can shorten the time of image preprocessing steps and it is good for a target area with a small range. Based on morphological processing, the interference of a large amount of redundant information is removed and the direction chain code is used to quickly extract the defect’s shape features to carry out the defect type identification. In order to ensure the adaptability of the system in a complex environment, the issues of adaptive dual threshold selection in edge detection, combined with the histogram concavity analysis, have been solved. In the rail area rapid locating method, the track defects are detected in real-time and the system has strict requirements for the detection time. If a crack is found on a track, its latitude and longitude coordinates are sent to the nearby base station.

The authors propose a mechanism to detect cracks on railway tracks in [27]. The study points out that ultrasonic metal detecting sensors are capable of detecting cracks with higher accuracy. Encoders and RF transmitters are used for crack detection, where a continuous flow of the current between the encoders shows that tracks are properly maintained. As long as the current remains continuous, the transmitter will broadcast RF signals. On the train’s engine, a receiver circuitry with a decoder is used. The receiver is linked to the train’s microprocessor, which regulates its operation. If a crack in the track occurs the current flow between the encoders will no longer be continuous. This prevents the transmitter from transmitting RF signals resulting in no signal being received by the locomotive’s receiver, causing the microcontroller to halt the train. Studies [16,21,22,23] performed experiments using wireless sensor networks and Bluetooth technology. Several different sensors were used to identify cracks on railway tracks; however, applying sensors and devices incurs a deployment cost, which makes such systems costly. In addition, faulty sensors require the replacement of the sensors which adds an extra cost to the system. Moreover, the maintenance of such systems requires skilled staff.

## 3. Proposed Research Methodology

This section contains the descriptions of the dataset collection strategy, the machine learning methods used for classification, and the proposed methodology.

### 3.1. Data Collection

**Figure 4:**
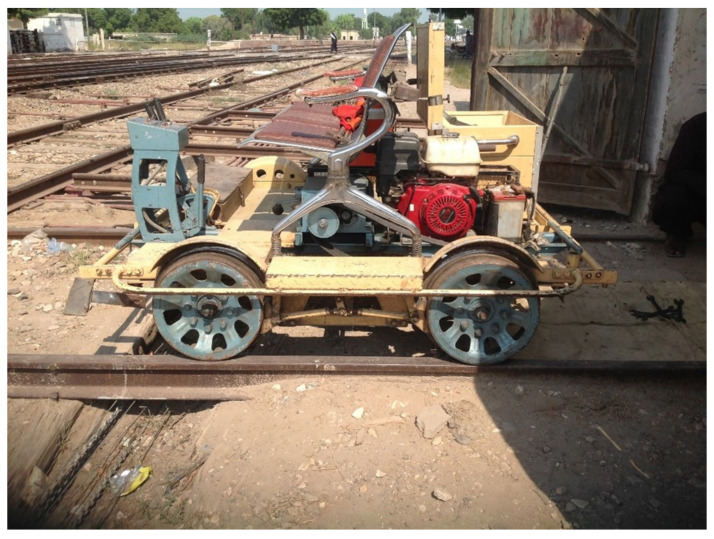
Mechanical railway cart used for data collection. The cart is driven by the engine that is manually controlled.

**Figure 5:**
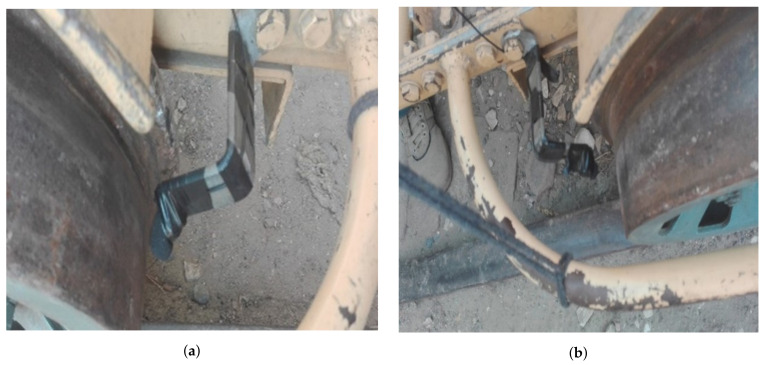
Pictures of wheels in contact with the track. (**a**) Assembly of microphone on left side of the mechanical cart; (**b**) Assembly of microphone on right side of the mechanical cart.

For automatic railway track fault detection, the dataset has the first and foremost importance. For dataset collection, a mechanical cart provided by Pakistan Railways Khanpur district Rahim Yar Khan station’s authorities was used as shown in Figure 4. For dataset collection, an onsite setup was implemented at the railway station in Khanpur. Two microphones were mounted at the safest maximum closest distance (1.75 inches) from the point of contact of the wheel and track. Microphones were attached to the right and left sides of the cart for data collection. Figure 5 shows the assembly of microphones attached on the left and right sides of the cart. The mechanical cart was driven by a generator that keeps the cart engine in operation with an average speed of 35 km per hour. The geographical location was not attached to the collected audio data and is left for future work.

Two microphones, ECM-X7BMP Unidirectional electric condensers, supplied with a 3-pole locking mini plug, were embedded on the left and right wheels of the railway cart. These microphones have a sensitivity of −44.0 ± 3 dB, while the output impedance is 1.2 kΩ± 30%. Other parameters of the microphone are provided in Table 1.

**Figure 6:**
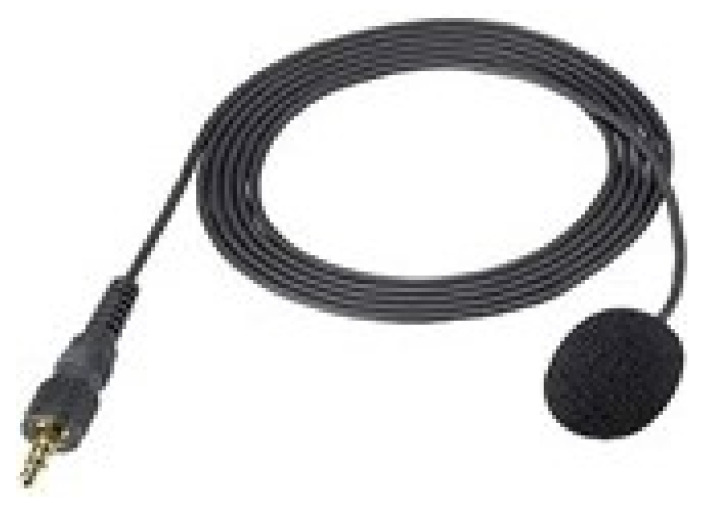
ECM-X7BMP type microphone [28].

Recordings took place for both microphones situated in two different positions. The microphone was set to turn on simultaneously to record on a single trigger button. Data were recorded as a “.wav” file with 16-bit audio format. Figure 6 shows the picture of the Sony ECM-X7BMP used for data collection. A metal strip was designed which was used to tightly hold the microphone at one end, while the other end was screwed firmly to the cart as shown in Figure 5a. The microphone diaphragm was protected from air gusts by foam or fur. Wind or breathing might produce loud pops in the audio signal if there is no windscreen. A Foam windshield was used to reduce cart vibrations to prevent its transition to the microphone because the Foam windshields are usually the first line of defense against wind noise. An open-cell foam cover around the microphone will disperse and diminish the acoustical energy of the wind hitting the mic capsule, reducing that low-end vibration. These need to be streamlined so that the wind flows around it rather than into it. Little vibration was present uniformly in the whole audio signal. It was present in normal track sounds and faulty track sounds as well so it had no impact on faulty signals. Before the air gusts interact with the microphone diaphragm, the windscreens broke them up.

During the data collection, a total of 720 audio recordings were made using the above-described setup where each file had a duration of 17 s. A sampling frequency of 22,050 Hz was used for data collection. Subsequently, the recordings were then labeled manually to structure the dataset. The collected audio recordings were then segmented into 758 frames using a window length of 1024 with a hop size of 512.

The experiments were carried out using aPython Jupyter notebook by using Google Colab services. Librosa was used for feature extraction (MFCC features). For Machine learning models, the sci-kit-learn library was used while for deep learning models, the Tensor Flow library was used.

### 3.2. Proposed Methodology for Track Fault Detection

**Figure 7:**
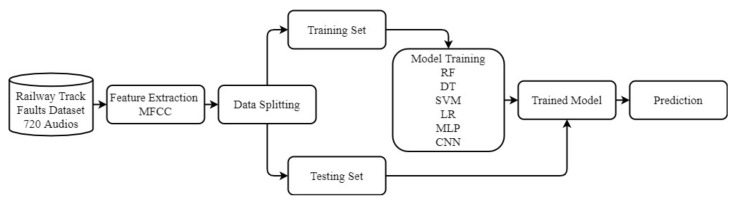
Architecture of the proposed methodology for faulty track detection comprising data collection, MFCC feature extraction, and training and testing the models.

Figure 7 shows the architecture of the proposed methodology for detecting three types of railway tracks. The captured audio data were used for faulty track detection. For this purpose, acoustic features from the audio data were used to train the machine and deep learning algorithms. This study used 40 Mel-frequency cepstral coefficients (MFCC) per frame of the audio data. This ended up with a matrix ‘M’ of 758 rows and 40 columns where 758 rows represent the frames, and 40 columns represent the MFCC values.

MFFC implementation steps are mentioned below [29]:

- Shorten the signal by framing it in brief frames;
- Calculate the power spectrum period gram estimate for each frame;
- Total the energy in each filter, apply the Mel-filter bank to the power spectra;
- Add all of the filter bank energies and find the logarithm;
- Take the log filter bank energies’ DCT;
- DCT coefficients 1–40 should be kept and the rest should be discarded.

The approximation of Mel from physical frequency can be expressed in Equation (1). More details are provided in the study [30,31,32], which worked on MFFC.

**Figure 8:**
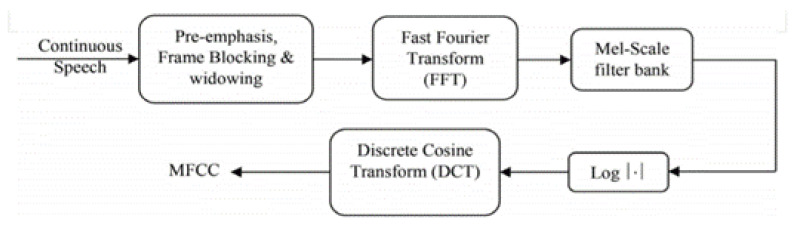
Five steps to extracting MFCC features [33]. It shows the steps followed to extract MFCC features that are used for the training and testing of the machine learning models.

The process of obtaining MFCC features is displayed in Figure 8. MFCC is based on signal disintegration with the help of a filter bank. The MFCC gives a discrete cosine transform (DCT) of a real logarithm of the short-term energy displayed on the Mel frequency scale. The formula used to calculate the Mel for a frequency is given by:

(1) $$mel\left(f\right)=2592\times log10\left(1+\frac{f}{700}\right),$$

where mel(f) is the frequency in mels and *f* is the frequency in Hz.The final feature vector space ‘F’ of size 40 is obtained as follows:

(2) $$F=\left[\frac{1}{N}\sum_{i=1}^{758}a_{i1},\frac{1}{N}\sum_{i=1}^{758}a_{i2},\frac{1}{N}\sum_{i=1}^{758}a_{i3},\ldots ,\frac{1}{N}\sum_{i=1}^{758}a_{i40}\right],$$

where *i* is the *i*th frame and *N* is the total number of frames, that is, 758. Subsequently, the *F* for all audio recordings (Normal track, Superelevation, Wheel burnt) was calculated and then labeled manually to structure the dataset and *F* was then used in the experimental setup. In the presence of an expert (Mechanical Engineer) from Pakistan Railway, faults on the tracks were identified and labeled. Subsequently, all audio recordings related to specific faults were stored in separate folders.

(3) $$M={\left(\begin{array}{ccccc} a_{11} & a_{12} & a_{13} & \ldots & a_{1C}\\ a_{21} & a_{22} & a_{23} & \ldots & a_{2C}\\ \ldots & \ldots & \ldots & \ldots & \ldots .\\ a_{(R-1)1} & a_{(R-1)2} & a_{(R-1)3} & \ldots & a_{(R-1)C}\\ a_{R1} & a_{R2} & a_{R3} & \ldots & a_{RC}, \end{array}\right)}_{R\times C}$$

where *R* is the number of rows, *C* is the number of columns and $a_{ij}$ is the MFCC coefficient value of *i*th frame *j*th MFCC coefficient value. The MFCC uses a quasi logarithmic spaced frequency scale which is close to the human auditory system. Matrix *M* indicates the features after performing all the steps shown in Figure 8 and the *M* matrix is used to classify the sample into one of the categories considered in this study. The matrix *M* consists of the extracted MFCC features for one sample, which means that each sample of railway track cracks (superelevation, wheel burn, etc.) has an *M* matrix containing its features. Each element in the matrix *M* is an MFCC coefficient value for a particular frame from a particular class of crack. These features were used to both train and test the machine learning algorithms.

**Figure 9:**
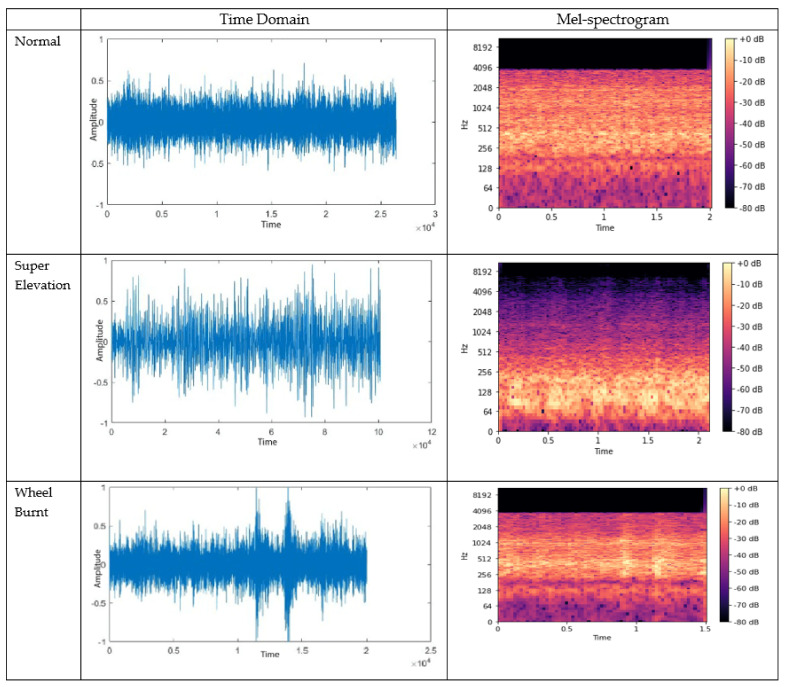
Normal, super elevation and wheel burn signals in time domain and MFCC. Mel-spectrogram shows clear difference in the signals for different faults.

Figure 9 shows the time domain and the Mel spectrogram plots of a normal track, wheel burn, and superelevation acoustic signals. The visual difference can be seen among these three types of track sounds. From Figure 9, the sound intensity distribution across different frequency ranges in the Mel-spectrogram can be seen. As an example, in the distribution of noise in the frequency range of 64–256 Hz, the normal track sound contains around −30 to −60 dB and the superelevation intensity range in the same frequency range is −2 dB to −20 dB, while a track with wheel burn has a noise intensity of −20 dB to −72 dB in the same frequency range.

### 3.3. Experiment Setup

The faulty tracks were identified and verified by railway track inspection experts from Pakistan Railways. The cart was operated on the faulty tracks and recorded the audio signals for the following types of tracks:

- Normal (unfaulty) track sound;
- Wheel burn;
- Super elevation.

Wheel burns are caused by slipping of the driving wheel of locomotives on the rail surface. Wheel burns are generally noticed where there are steep gradients or where there are incidences of rain. Sometimes, in a case where the hauling power of the locomotive is not sufficient to carry the load of a train, wheel slip is noticed due to the rail temperature rising, resulting in the melting of the rail’s surface. These defects are known as wheel burns [34].

Superelevation is the rate of change in elevation (height) between the two rails or edges. This is normally greater where the railway is curved; raising the outer rail creates a banked turn, thus allowing vehicles to maneuver through the curve at higher speeds than would otherwise be possible if the surface was flat or level [35]. Wheel burn and superelevation are among the common factors responsible for railway derailing accidents [36,37].

There are many railway track faults that exist, such as broken rails and welds, track geometry, wide gauges, missing nuts and bolts, disjoint, cracks, and so forth. However, in this study the authors only consider wheel burn and superelevation [38], the rest are left for future work. Experimental dataset collection was performed on the mainline where traffic normally runs, and for that moment, the allocated space only had these two issues present, so we performed our experiment on this line specifically.

The collected datasets were manually labeled by the railway tack engineer and were divided into two sets—training and testing. Different train–test splits were for training and testing. For fault detection and classification, machine learning models, such as SVM, LR, and deep learning classifiers, such as MLP and CNN, were exploited.

### 3.4. Mel Frequency Cepstral Coefficients

Mel frequency cepstral coefficients (MFCC) are often suggested for identifying monosyllabic words in continuously spoken sentences but not for speaker identification. MFCC computation is a replication of the human hearing system intending to artificially implement the ear’s working principle with the assumption that the human ear is a reliable speaker recognizer [39]. MFCC features are rooted in the recognized discrepancy of the human ear’s critical bandwidths with frequency filters spaced linearly at low frequencies and logarithmically at high frequencies being used to retain the phonetically vital properties of the speech signal. Speech signals commonly contain tones of varying frequencies, each tone with an actual frequency, *f* (Hz), and the subjective pitch is computed on the Mel scale. The Mel frequency scale has linear frequency spacing below 1000 Hz and logarithmic spacing above 1000 Hz. Pitch of 1 kHz tone and 40 dB above the perceptual audible threshold is defined as 1000 Mel, and used as a reference point [40]. Further details regarding MFCC and its use for audio signals analysis can be found in [31,32].

### 3.5. Supervised Machine Learning Models

This study performed experiments using SVM [41] and LR [42], as well as MLP [43] and CNN [44]. The performance of these models was optimized by fine-tuning several important hyperparameters. A list of the used hyperparameters is provided in Table 2.

#### 3.5.1. Logistic Regression

LR is one of the widely used linear models for data classification [45]. LR is used to explain the relationship between one dependent binary variable and one or more nominal, ordinal, interval, or ratio-level independent variables [42]. This study uses LR with five hyper-parameters which are tuned to optimize its performance. For the optimization, ‘saga’ algorithms with multinomial loss fit were used. All hyper-parameters for LR and their values are shown in Table 2.

#### 3.5.2. Support Vector Machine

SVM is a widely used model for both classification and regression. SVM draws the hyperplane to separate the data point with the best margin between the class boundaries [41]. The best hyper-plane is one that maximizes the margins from different data points. SVM has two main advantages: higher speed and better performance with a limited number of samples. This study used SVM with three hyper-parameters: ‘linear’ kernel, C regularization, and a random_state of 500.

#### 3.5.3. Random Forest

RF is a tree-based ensemble model that can be used for both classification and regression tasks. We used RF in this study for railway track fault classification [46]. RF combines numbers of decision trees under majority voting criteria which means that RF will generate decision trees and each tree predicts the target class [47]. Then RF will perform majority voting between decision tree predictions and the target class that is most predicted by decision trees will be the final prediction by RF. We can define it mathematically as:

(4) $$RF_{p}=mode\left\{dt_{1},dt_{2},dt_{3},\ldots ,dt_{n}\right\}\;\mathrm{OR}\;RF_{p}=mode\left\{\sum_{i=1}^{N}dt_{i}\right\}.$$

Here, $dt_{1},dt_{2},dt_{3},\ldots ,dt_{n}$ are the predictions by decision trees and $rf_{p}$ is the prediction by RF using majority voting. We used RF with the three hyperparameters shown in Table 2. The n_estimators we used with a value of 200, which means that RF will generate 200 decision trees for the prediction procedure and max_depth with a value of 50 which will restrict the decision trees to grow to a max 50 level depth to avoid complexity and over-fitting.

#### 3.5.4. Decision Trees

DT is a tree-based model used for both classification and regression tasks. DT consists of a root node and leaf nodes where the decision node has two or more branches while the leaf node represents a classification or decision [48]. To find the best split in the tree, DT used Entropy or Information Gain algorithms to construct the tree [49]. We used DT in this study with the two hyperparameters shown in Table 2. The max_depth hyperparameters we used had a value of 50, which will restrict the DT to grow to a max 50 level depth to avoid complexity and over-fitting.

### 3.6. Deep Learning Models

In addition to a machine learning classifier, deep learning models such as MLP are also used for detecting faulty railway tracks. Deep learning models have been utilized in a variety of tasks including indoor scene recognition, activity detection in smart homes, and events detection in smart cities, and so forth [50,51,52]. In addition to using single models, ensemble models tend to show a better performance, as reported in [53].

#### 3.6.1. Multilayer Perceptron

**Figure 10:**
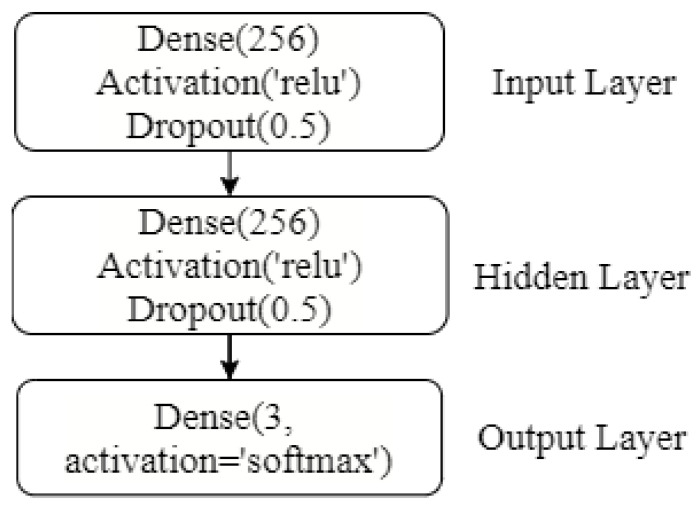
Architecture of MLP model designed for this study. Dense refers to a fully connected layer, activation is the activation function used while the dropout layer shows the neural dropout ratio used for optimization.

The multilayer perceptron is a widely used deep learning network for a variety of tasks including image processing, object detection, and NLP tasks, and so forth [43,44]. Figure 10 shows the architecture of MLP used for the experiments. It consists of three dense layers, three activation layers, and two dropout layers. The first two dense layers contain 256 neurons each, followed by the rectifier linear unit (ReLU) activation function and 0.5 dropouts. The output layer consists of three neurons to predict three classes of railway tracks and a softmax activation function.

#### 3.6.2. Convolutional Neural Network

**Figure 11:**
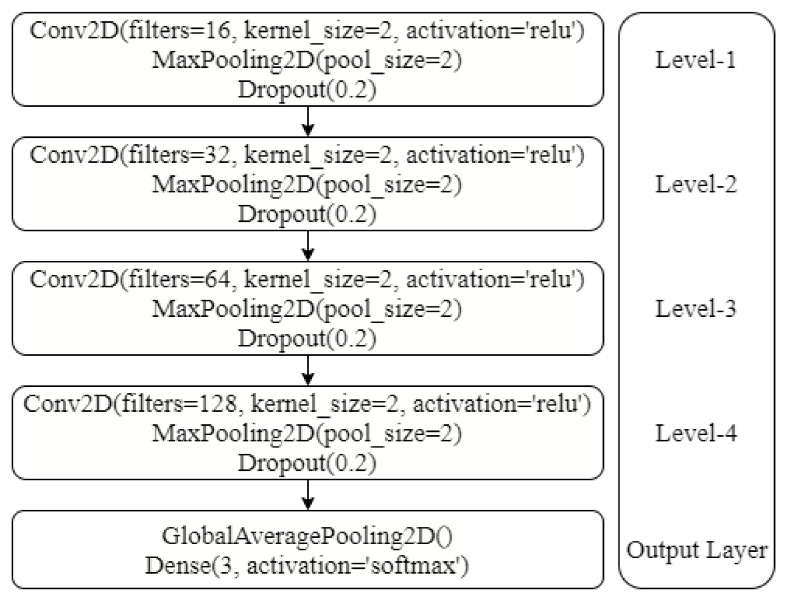
Architecture of CNN model used for experiments in this study. Conv2D shows the 2D convolutional layer with kernel size of 2 and max pooling layer with a pool size of 2. Dropout rate for neuron drop is 0.2 indicating 20% drop for optimization.

The architecture of the CNN is shown in Figure 11. The NN consists of four 2D convolutional (Conv2D) layers, four activation layers, four max-pooling layers, and four dropout layers. In the end, the output layer consists of one average pooling layer and one dense layer. Each Conv2D layer contains different filter sizes of 16, 32, 64, and 128 with an ReLU activation function and a kernel size of 2 × 2. Each Conv2D layer is followed by the ReLU activation function layer, 2 × 2 max-pooling 2D layer, and a dropout layer with a 0.2 dropout rate. In the end, the output layer has three neurons and an activation function to give the final prediction [54].

## 4. Results and Discussion

For the performance evaluation of the classification models, standard parameters, such as accuracy, precision, recall, and *F1* score, were used. Accuracy refers to the ratio of correctly predicted instances to the total predictions. Precision indicates the exactness of the classifier and considers the number of true positives (*TP*) to *TP* and false positives (*FP*). Recall, also known as sensitivity, takes into account *TP* and the summation of *TP* and false negatives (*FN*). Precision and recall alone can be misleading, so often the h1 score is used to indicate the performance of the models. The *F*1 score considers both precision and recall and provides a value between 0 and 1. The *F1* score, often called the F-measure, is defined as the harmonic mean of precision and recall.

Mathematical equations for accuracy, precision, recall, and F1 scores are given here:

(5) $$Accuracy=\frac{TP+TN}{TP+TN+FP+FN};$$

(6) $$Precision=\frac{TP}{TP+FP};$$

(7) $$Recall=\frac{TP}{TP+FN};$$

(8) $$F1=2\times \frac{Precision\times Recall}{Precision+Recall}.$$

Experiments were performed using the selected models with MFCC features from the collected audio data with different ratios of train–test splits including 60:40, 70:30, 80:20, and 90:10 for train and test, respectively. The objective of using multiple train–test splits was to analyze the performance of the machine learning and deep learning models when the amount of training data was changed. Table 3 shows the classification results obtained using a 60–40 train–test split. RF and DT achieved the highest accuracy of 0.97 each, followed by LR. The lowest accuracy was by MLP, which was 0.68, with a large difference in precision and recall.

Table 4 shows the performance evaluation metrics when 70% data were used for training. Results indicate that RF outperforms both machine learning and deep learning models in terms of accuracy, precision, recall, and F1 score. The 0.96 accuracy of DT is marginally lower than RF, followed by LR with a 0.94 accuracy. Other parameters for RF, DT, and LR are in the conformation of accuracy which shows a good fit of these models to the training data.

Using an 80–20 train-test split, the machine learning classifiers sustain their performance and there is no improvement in the classification accuracy, as shown in Table 5. A marginal difference in the accuracy is observed in RF and DR while the accuracy of LR is improved from 0.74 to 0.76 when the training data are increased. On the other hand, SVM has a reduced accuracy of 0.77 compared to 0.79 with a 70–30 train–test split. In the end, results using a 90–10 train–test split are given in Table 6, which indicate that LR, RF, and DT have accuracy scores of 0.97, 0.96, and 0.94, respectively, and are among the best performers. Precision, recall, and F1 scores of these classifiers are very similar to the accuracy, indicating good fits for these classifiers.

Traditionally, SVM works well with unstructured and semi-structured data and performs well with text data. Based on the geometrical properties, it shows a better performance with a small number of features for a small number of training samples. However, it is not the case here, as the data used for experiments are structured with a large number of features. Therefore, LR shows a better performance with an accuracy of 0.97 for detecting normal, superelevation, and wheel burnt railway tracks. The F1 score has a close resemblance to accuracy, which indicates that the model is a good fit.

For illustrating the good fit of the models and proving that the models are not overfitted on the data, Table 7 is provided. It shows the results of all the models on the training data used for the experiments. So it provides the training accuracy for the different splits of data used for training.

Generally, deep learning architectures show a superior performance as compared to the machine learning models. Deep learning models are better at understanding the complex relationships found in the data and show good results. However, the performance of MLP and CNN is comparatively poor for the experiments conducted in this study. The primary reason is the number of samples used for training the deep learning models. MLP and CNNs show better results when trained on large datasets containing thousands of samples for each class. However, the dataset used in this study contains 720 samples in total which is not enough to get a good fit for deep learning models. Consequently, the performance of the deep learning [55] models is poor. The architecture of the CNN is further optimized using different levels of CNN after evaluating each layer. For this purpose, CNN layers are presented in a stacking manner as shown in Figure 11. After levels 3 and 4, there is no change in the accuracy, so the execution is stopped as further optimization is not possible. The results of the CNN at each level are provided in Table 8.

**Figure 12:**
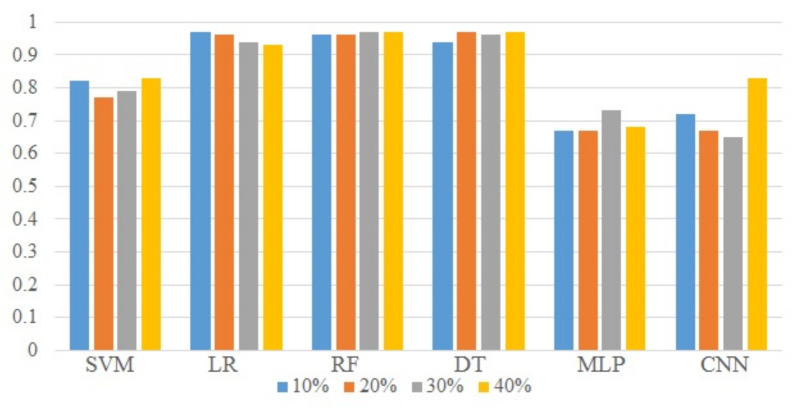
Classification accuracy using different train-test splits.

Figure 12 presents the accuracy, precision, recall, and F1 score of all the classifiers used in the study. It indicates that CNN has large fluctuations in accuracy when the amount of training data is changed. Traditionally, too little training data leads to poor approximation and the model will underfit the small training dataset. Conversely, an under-constrained model will likely overfit the training data. For both underfit and overfit cases, the result is poor performance. Fluctuations in the performance of deep learning models are attributed to a smaller change in the training data size. For obtaining good results from deep learning models using supervised learning, further experiments are needed to estimate the amount of data required to approximate the underlying mapping function and the amount of test data needed to determine the performance. Results reported in Table 3, Table 4, Table 5 and Table 6 show the test accuracy. For performance evaluation, k-fold cross-validation is performed as well.

Cross-validation results given in Table 9 indicate that DT and RF are the best performers for the task at hand with 0.96 accuracy each with a standard deviation of 0.02 and 0.04, respectively. MLP shows the worst performance among all the used classifiers with 0.66 accuracy. On average, machine learning classifiers perform better than deep learning models owing to the amount of data used for the experiments.

## 5. Conclusions and Future Work

Railway track health monitoring is important for smooth railway operation. The lack of a robust track fault detection mechanism may lead to accidents and losses in terms of assets, time, and passengers; hence proper and timely maintenance should be implemented by detecting the causes in time to avoid disasters. The existing traditional railway cart for track inspection requires manual inspection, which is mainly based on human judgment for track fault detection in many underdeveloped countries. A smart railway cart is proposed for detecting cracks on railway tracks automatically by way of acoustic analysis. The proposed approach has been investigated in a real environment and acoustic data were collected and different machine learning and deep learning algorithms were applied to compare them based on accuracy. Different train–test splits were used to evaluate the performance of machine learning algorithms and the results indicate that the best results are obtained using RF and DT with an accuracy of 97%. Further investigation in the future will include enhancing the dataset in different terrains and incorporate other sensors such as a gyroscope, a seismic sensor, and an optical sensor to further improve the performance and robustness. Moreover, the sensor deployment on the locomotive is also under consideration. Furthermore, in future, the cart will be capable of recording the geographical location for each audio recording and the location of the track fault will be provided along with the fault type.

## Figures and Tables

**Table 1:** Important parameters of Sony ECM-X7BMP microphone.

Parameter	Value
Sensitivity	−44.0 ± 3 dB
Output impedance	1.2 kΩ ± 30
Dynamic range	88 dB
Signal-to-Noise Ratio	62 dB
Max. input sound pressur elevel	120 dB PSL
Direction	Unidirectional
Connectivity	Wired
Operating voltage	5.0 V

**Table 2:** Hyperparameters that are fine-tuned to optimize the performance of the machine learning models.

Algorithm	Hyperparameters
LR	solver = saga, C = 2.0, max_iter = 100, penalty = ‘l2’, multi_clas = multinomial
SVM	kernel = ‘linear’, C = 2.0, random_state = 500
RF	n_estimators = 200, max_depth = 50, random_state = 2
DT	max_depth = 50, random_state = 2
MLP	Input layer, Hidden layer, Output layer, optimizer = adam, Dropout = 0.5
	loss = categorical_crossentropy, activation= ReLU, Softmax, epoches = 200
CNN	Conv2D (filter = 16, 32, 64, 128, kernel = 2 × 2), maxpooling2D = 2 × 2,
	optimizer = adam, loss = categorical_crossentropy, Dropout = 0.5, epoches = 200

**Table 3:** Results of machine learning and deep learning classifiers for 60–40 split.

Classifier	Accuracy	Precision	Recall	F1 Score
SVM	0.83	0.84	0.82	0.82
LR	0.93	0.93	0.93	0.93
RF	0.97	0.97	0.97	0.97
DT	0.97	0.97	0.97	0.97
MLP	0.68	0.51	1.0	0.67
CNN	0.83	0.89	0.82	0.82

**Table 4:** Results of machine learning and deep learning classifiers using 70–30 train-test split.

Classifier	Accuracy	Precision	Recall	F1 Score
SVM	0.79	0.82	0.78	0.77
LR	0.94	0.94	0.94	0.94
RF	0.97	0.97	0.97	0.97
DT	0.96	0.97	0.96	0.96
MLP	0.73	0.75	0.73	0.72
CNN	0.65	0.49	0.65	0.55

**Table 5:** Results of machine learning and deep learning classifiers using 80–20 train–test split.

Classifier	Accuracy	Precision	Recall	F1 Score
SVM	0.77	0.84	0.77	0.74
LR	0.96	0.96	0.96	0.96
RF	0.96	0.96	0.96	0.96
DT	0.97	0.97	0.97	0.97
MLP	0.67	0.50	0.67	0.56
CNN	0.67	0.50	0.67	0.56

**Table 6:** Results of machine learning and deep learning classifiers with 90–10 train–test split.

Classifier	Accuracy	Precision	Recall	F1 Score
SVM	0.82	0.86	0.82	0.81
LR	0.97	0.97	0.97	0.97
RF	0.96	0.96	0.96	0.96
DT	0.94	0.95	0.94	0.94
MLP	0.67	0.50	0.67	0.56
CNN	0.72	0.85	0.72	0.66

**Table 7:** Results of training accuracy for all models.

Classifier	90%	80%	70%	60%
SVM	0.76	0.76	0.79	0.78
LR	0.90	0.89	0.94	0.88
RF	0.96	0.96	1.00	0.97
DT	0.97	0.97	1.00	0.97
MLP	0.66	0.66	0.74	0.65
CNN	0.71	0.66	0.67	0.83

**Table 8:** Results of CNN at different level of architecture.

CNN	Accuracy	Precision	Recall	F1 Score
Level 1	0.55	0.49	0.55	0.51
Level 2	0.68	0.53	0.55	0.55
Level 3	0.68	0.53	0.69	0.58
Level 4	0.68	0.53	0.69	0.58

**Table 9:** Results for both machine and deep learning models using k-flod cross-validation.

Classifier	Accuracy (Std. Dev.)
SVM	0.77 (±0.07)
LR	0.90 (±0.07)
RF	0.96 (±0.04)
DT	0.96 (±0.02)
MLP	0.66 (±0.05)
CNN	0.72 (±0.07)

## Data Availability

Not applicable.
